# Novel Anti-CRISPR-Assisted CRISPR Biosensor for Exclusive Detection of Single-Stranded DNA (ssDNA)

**DOI:** 10.1021/acssensors.4c00201

**Published:** 2024-03-05

**Authors:** Qiaoqiao Ci, Yawen He, Juhong Chen

**Affiliations:** † Department of Biological Systems Engineering, Virginia Tech, Blacksburg, Virginia 24061, United States; ‡ Department of Bioengineering, University of California, Riverside, Riverside, California 92521, United States

**Keywords:** CRISPR-based biosensor, Cas12a (cpf1) nuclease, anti-CRISPR proteins, AcrVA1, single-stranded DNA (ssDNA)

## Abstract

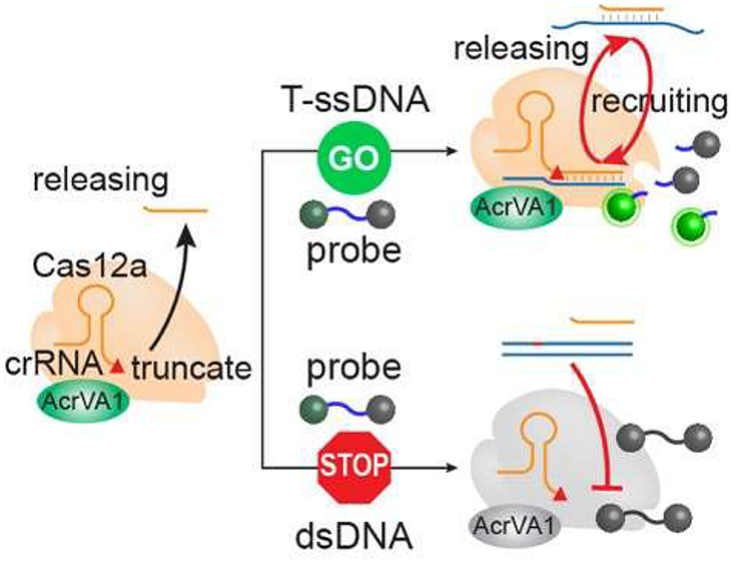

Nucleic acid analysis plays an important role in disease diagnosis and treatment. The discovery of CRISPR technology has provided novel and versatile approaches to the detection of nucleic acids. However, the most widely used CRISPR-Cas12a detection platforms lack the capability to distinguish single-stranded DNA (ssDNA) from double-stranded DNA (dsDNA). To overcome this limitation, we first employed an anti-CRISPR protein (AcrVA1) to develop a novel CRISPR biosensor to detect ssDNA exclusively. In this sensing strategy, AcrVA1 cut CRISPR guide RNA (crRNA) to inhibit the cleavage activity of the CRISPR-Cas12a system. Only ssDNA has the ability to recruit the cleaved crRNA fragment to recover the detection ability of the CRISPR-Cas12 biosensor, but dsDNA cannot accomplish this. By measuring the recovered cleavage activity of the CRISPR-Cas12a biosensor, our developed AcrVA1-assisted CRISPR biosensor is capable of distinguishing ssDNA from dsDNA, providing a simple and reliable method for the detection of ssDNA. Furthermore, we demonstrated our developed AcrVA1-assisted CRISPR biosensor to monitor the enzymatic activity of helicase and screen its inhibitors.

Genetic information in all forms of life is carried by DNA, of which there are two forms, single-stranded DNA (ssDNA) and double-stranded DNA (dsDNA).^1,2^ ssDNA is a major signal of replication distress that activates cellular checkpoints, as well as a potential source of genome instability as it is susceptible to mutation and recombination.^3,4^ Compared to dsDNA, it is more important to analyze ssDNA for many analytical applications, such as molecular biology, clinical diagnostics, and medical research.^5−7^ Most traditional methods to detect ssDNA rely on the polymerase chain reaction (PCR) or probe hybridization but suffer from several limitations, such as nonspecific amplification, poor hybridization, and/or complicated detection processes.^8−10^ To date, it is still challenging to distinguish ssDNA from dsDNA with high sensitivity and specificity.

CRISPR, clustered regularly interspaced short palindromic repeats technology, has been explored as a novel tool to detect nucleic acids.^11−13^ Especially, the CRISPR-Cas12 system has been widely explored to detect various DNA-related targets, including viruses, bacteria, and cancer biomarkers.^14,15^ The CRISPR-Cas12a system comprises two essential components: a single-strand CRISPR RNA (crRNA) and a CRISPR Cas12a nuclease. The crRNA is used to navigate the Cas12a nuclease to cut a specific DNA sequence, and the Cas12a nuclease acts like scissors to cut DNA. The CRISPR-Cas12a system initiates DNA cleavage by identifying the specific protospacer adjacent motif (PAM), which is TTTN for CRISPR Cas12a. After the crRNA undergoes hybridization with the target DNA, Cas12a exhibits its *cis*-cleavage activity, cleaving the DNA into two parts. Furthermore, the Cas12a activation leads to nonspecific *trans*-cleavage of degrading nearby ssDNA into smaller fragments.^16,17^ This *trans*-cleavage activity offers the CRISPR-Cas12a system to be developed as a novel biosensing technique for the detection of DNA-of-interests in the fields of clinical diagnosis,^18−22^ environmental monitoring,^23−25^ and food safety.^26−28^

Although the CRISPR-Cas12a systems have been widely applied to detect DNA-based targets, none of these systems have the ability to differentiate ssDNA from dsDNA. To address this issue, we introduced the anti-CRISPR proteins in the CRISPR-Cas12 system to detect ssDNA. Anti-CRISPR proteins are small proteins (∼50–200 amino acids in length) that can inhibit the CRISPR cleavage activity.^25,29,30^ Usually, the anti-CRISPR proteins are evolved in phage to inactivate CRISPR nucleases and enable phage replication.^31,32^ The anti-CRISPR proteins for Cas12a are known as type V-A, named as AcrVAs. A total of 5 AcrVAs have been reported, but only 3 (AcrVA1, AcrVA4, and AcrVA5) showed the ability to inhibit the enzymatic activity of the CRISPR-Cas12a system through distinct mechanisms. In this study, we first introduced the anti-CRISPR type V-A1 protein (AcrVA1) into the CRISPR-Cas12a biosensor to detect ssDNA exclusively. Our developed AcrVA1-assisted CRISPR biosensor developed here can precisely detect ssDNA exclusively, which has been further demonstrated to detect the enzymatic activity of helicase and screen its inhibitors.

Prior to our detection, we prepared two Cas12a nucleases (LbCas12a and AsCas12a) that had slight differences in the crRNA sequence to detect DNA and three anti-CRISPR proteins that can inhibit the CRISPR-Cas12a biosensor by different mechanisms (Figure S1). The detection mechanism of ssDNA and dsDNA using the CRISPR-Cas12a biosensor is shown in Figure 1a. The crRNA is highly target-specific, serving as a recognition element to target DNA. The specific recognition events can then be transduced and amplified by the *trans*-cleavage activity of Cas12a to cleave ssDNA-FQ probes (the fluorophore and quencher are linked by ssDNA). The resulting fluorescence signals from the cleaved probes can be used to determine the concentration of target DNA. According to a previously published work, the concentrations of Cas12a (5 nM), crRNA (6.25 nM), and ssDNA-FQ probes (10 nM) were used in this study.^28^ All reactions were performed at 37 °C. Consistent with the previous studies, the CRISPR-Cas12a biosensor can be activated by both ssDNA and dsDNA to cleave the ssDNA-FQ probes, resulting in strong fluorescent signals (Figure 1b). There is no significant difference in the fluorescence of ssDNA and dsDNA. Conclusively, the ssDNA and dsDNA cannot be distinguished using either of the two CRISPR-Cas12a biosensors.

**Figure 1:**
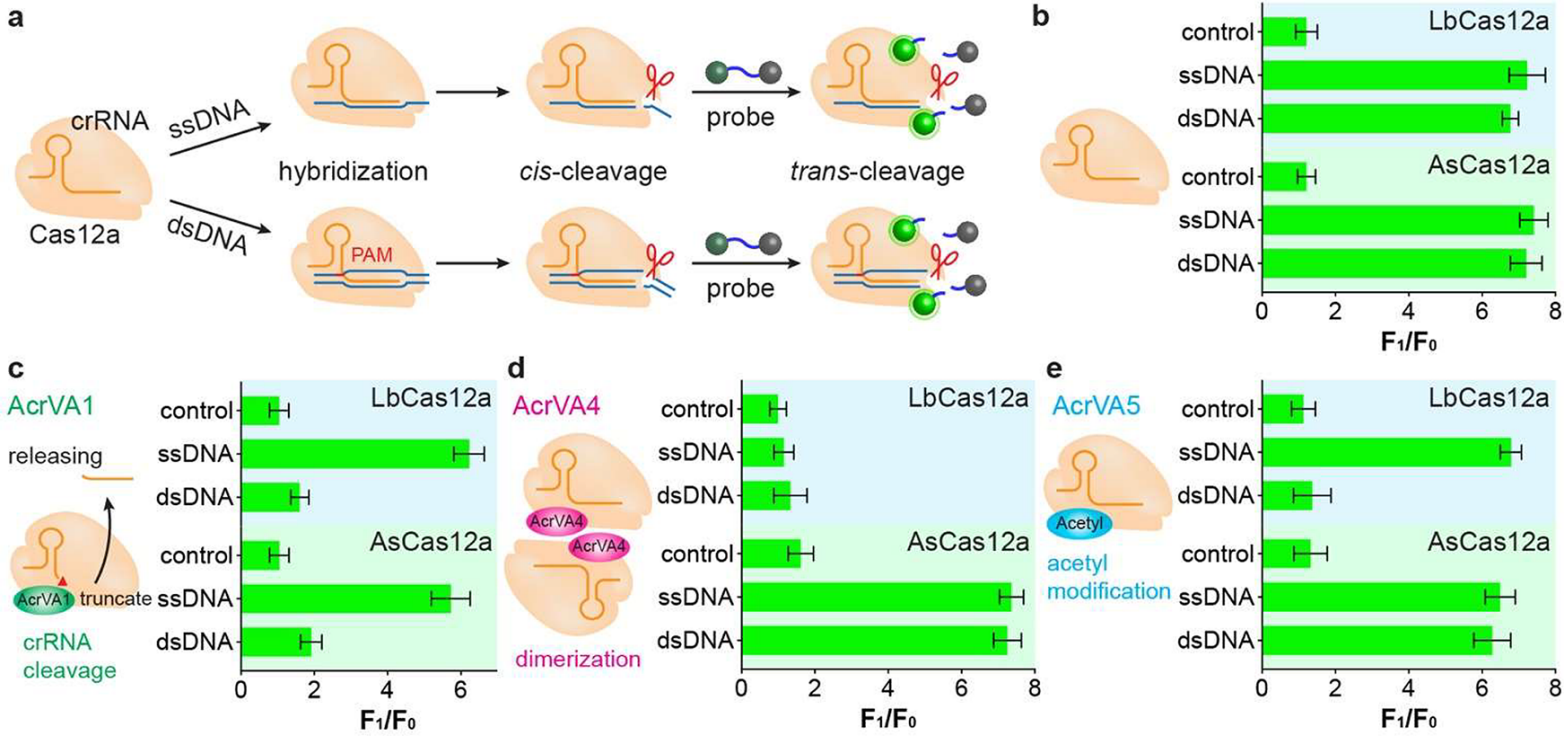
(a) Schematic illustration of the CRISPR-Cas12a biosensor to detect ssDNA and dsDNA. (b) The relative fluorescence responses of the CRISPR-Cas12a biosensor to detect ssDNA and dsDNA. (c, d) The inhibition effect of AcrVA1, AcrVA4, and AcrVA5 on the performances of the CRISPR-Cas12a biosensor to detect ssDNA and dsDNA, respectively. All experiments were performed with at least three replicates, and the error bars represent the standard deviation. F_1_ represents the florescence intensity at the end point, while F_0_ represents the initial fluorescence intensity.

Next, we investigated whether anti-CRISPR proteins could assist the CRISPR-Cas12a biosensor in detecting ssDNA exclusively. Anti-CRISPR proteins are ∼50–200 amino acids in length generated by viruses that can inhibit the activity of CRISPR systems.^25,29,30^ The anti-CRISPR type V-A proteins (AcrVAs) can inhibit the activity of Cas12a nucleases. A total of 5 AcrVAs have been reported, but only 3 (AcrVA1, AcrVA4, and AcrVA5) showed the ability to inhibit the cleavage activity of Cas12a nucleases, each employing distinct mechanisms.^33,34^ Briefly, AcrVA1 cleaves the crRNA in the Cas12a-crRNA complex; AcrVA4 homodimer restrains the conformational changes necessary for the formation of crRNA-DNA heteroduplex, and AcrVA5 functions as an acetyltransferase.^33,34^ All three AcrVAs were introduced to the CRISPR-Cas12a biosensor to detect ssDNA and dsDNA, respectively. As shown in Figure 1c–e, we compared the effects of the three AcrVAs on the performances of the CRISPR-Cas12a biosensor to detect ssDNA and dsDNA. AcrVA1 has the ability to distinguish ssDNA from dsDNA for both LbCas12a and AsCas12a (Figure 1c). No matter whether it is LbCas12a or AsCas12a, AcrVA4 cannot be used to detect ssDNA (Figure 1d). Interestingly, AcrVA5 can be applied to detect ssDNA for LabCas12a, but not for AsCas12a (Figure 1e). This can be explained that AsCas12a, different from LbCas12a, contains an ancestral helical bundle that can escape the inhibiting attack by AcrVA4 and AcrVA5.^31^ As a result, only AcrVA1 can allow both CRISPR-LbCas12a and CRISPR-AsCas12a biosensors to detect ssDNA exclusively.

We further studied the effect of AcrVA1 on the CRISPR-Cas12a biosensor to detect ssDNA (Figure 2). AcrVA1 is a highly effective and broad-spectrum inhibitor of the CRISPR-Cas12a system by truncating the predesigned crRNA. The addition of target ssDNA (T-ssDNA), which is complementary to the crRNA, can recruit the truncated crRNA fragment to the CRISPR-Cas12a complex, resulting in the DNA cleavage recovery of the CRISPR-Cas12a system (Figures 2a and S2). As a result, the recovered CRISPR-Cas12a system possesses both *cis*-cleavage of T-ssDNA and *trans*-cleavage of ssDNA-FQ probes again. However, the nontarget ssDNA (NT-ssDNA) and target dsDNA fail to recruit the truncated crRNA fragment back to the Cas12a-crRNA complex (Figure 2a). The incomplete Cas12a-crRNA complex cannot trigger *cis*-cleavage of NT-ssDNA or dsDNA and causes the loss of the ability *trans*-cleavage of the ssDNA-FQ probes to produce fluorescence signals.^35^

**Figure 2:**
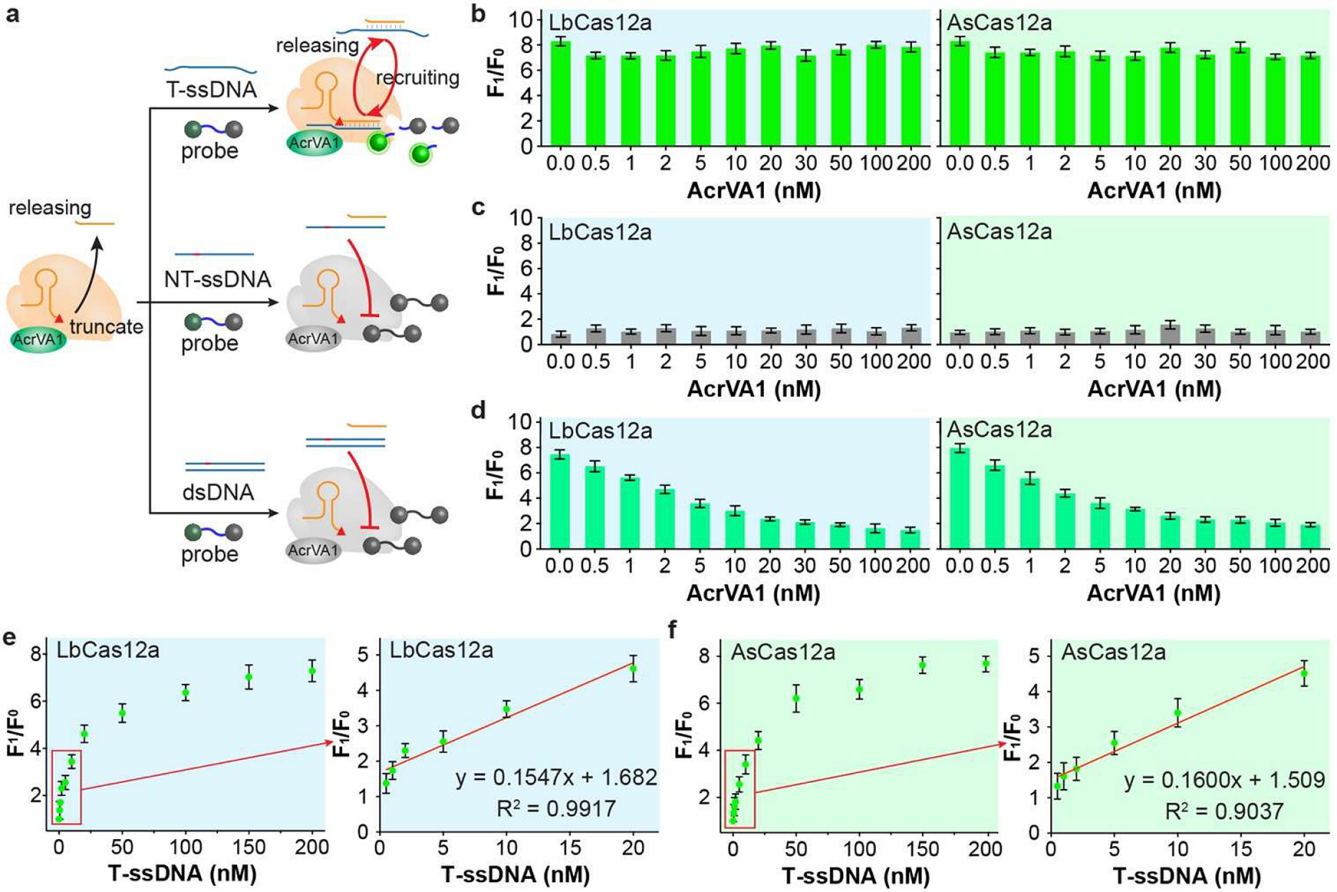
(a) Schematic illustration of the AcrVA1-assisted CRISPR-Cas12a biosensor to detect T-ssDNA, NT-ssDNA, and dsDNA. (b–d) Effect of AcrVA1 concentration on the performance of the CRISPR-Cas12a biosensors to detect T-ssDNA, NT-ssDNA, and dsDNA, respectively. (e, f) Detection sensitivity of T-ssDNA using the AcrVA1-assisted CRISPR-Cas12a biosensor. All experiments were performed with at least three replicates, and the error bars represent the standard deviation.

We next investigated the effect of the AcrVA1 concentration on the performance of the CRISPR-Cas12a biosensor to detect T-ssDNA, NT-ssDNA, and dsDNA (Figure 2b–d). In this study, AcrVA1 concentrations ranging from 0 to 200 nM were added to the CRISPR-Cas12a biosensor when detecting T-ssDNA, NT-ssDNA, and dsDNA, respectively. For T-ssDNA, the AcrVA1 concentration did not alter the fluorescence intensities for both CRISPR-LbCas12a and CRISPR-AsCas12a biosensors (Figure 2b). In the case of NT-ssDNA, given the lack of complementary pairing between NT-ssDNA and crRNA, the CRISPR-Cas12a biosensor did not generate a response signal in both the absence and presence of the AcrVA1 (Figure 2c). When detecting dsDNA, the fluorescence intensity of the CRISPR-Cas12a biosensor progressively decreased with the increase of AcrVA1 concentration and leveled off at 30 nM (Figure 2d). Thus, we chose AcrVA1 at the concentration of 30 nM in subsequent experiments. Although there are some variations in the normalized fluorescence intensity with the increase of the AcrVA1 concentration, there are significant differences between T-ssDNA and the other two (NT-ssDNA and dsDNA).

After determining the AcrVA1 concentration, we evaluated the detection sensitivity of T-ssDNA using novel AcrVA1-assisted CRISPR-Cas12a systems. In this study, T-ssDNA at various concentrations was added into the novel detection platform (named AcrVA1-assisted CRISPR-Cas12a system). The fluorescence spectra of T-ssDNA at different concentrations were recorded (Figure S3). Incorporating AcrVA1 into the CRISPR-LbCas12a system, the fluorescence intensity increased with the increase of T-ssDNA, and a good linear relationship was observed between the fluorescence intensity and T-ssDNA concentration in the range from 0.5 to 20 nM (Figure 2e). The regression equation is expressed as y = 0.1547x + 1.682 (R^2^ = 0.9117), where y and x represent the fluorescence intensity and T-ssDNA concentration, respectively. The detection limit is calculated to be 0.12 nM, obtained by the average signal of the control group plus three times the standard deviation. Similarly, the AcrVA1-assisted CRISPR-AsCas12a system also exhibited an excellent ability to detect T-ssDNA (Figure 2f). The corresponding equation is y = 0.1600x + 1.509 (R^2^ = 0.9037), and the detection limit is determined to be as low as 0.25 nM. Therefore, our developed novel AcrVA1-assisted CRISPR-Cas12a biosensor has shown great potential for the detection of ssDNA with high sensitivity.

Inspired by these exciting results, we investigated whether our novel AcrVA1-assistant CRISPR biosensor could be used to monitor dsDNA unwinding catalyzed by DNA helicase. Helicase is an essential enzyme for cell growth and proliferation and has been reported to be closely associated with several genetic diseases. In this study, UvrD helicase, also known as *Escherichia coli* helicase II, was chosen as a model analyte, in which the enzyme is capable of unwinding dsDNA into ssDNA.^36^ The UvrD helicase plays a critical role in the replication, recombination, and repair of mismatched base pairs.^37^ As shown in Figure 3a, our developed AcrVA1-assisted CRISPR biosensor can be activated by helicase-unwinded ssDNA to generate fluorescence signals. However, the entire dsDNA cannot trigger the CRISPR-Cas12a biosensor in the presence of AcrVA1 at a high concentration, as demonstrated in Figure 2d. Thus, our novel AcrVA1-assisted CRISPR biosensor can be applied to monitor the enzymatic activity of UvrD helicase. The fluorescence intensities at the wavelength of 525 nm increased significantly with the increase of helicase concentration (Figure S4). For the AcrVA1-assisted CRISPR-LbCas12a biosensor, a linear correlation was observed between the relative fluorescence intensity and the UvrD helicase concentration in the range of 0.25 to 3 μg/mL (Figure 3b). The correlation satisfies the equation y = 1.136x + 1.213 (R^2^ = 0.9346), and the detection limit was calculated to be 0.12 μg/mL. Regarding AsCas12a, a good linear relationship (y = 1.168x + 01.248, R^2^ = 0.9166) was observed as the concentrations of UvrD helicase increased from 0.25 to 2.0 μg/mL, and the detection limit was calculated to be 0.26 μg/mL (Figure 3b). These results indicate that the novel AcrVA1-assisted CRISPR biosensor exhibited an excellent sensing ability toward the UvrD helicase.

**Figure 3:**
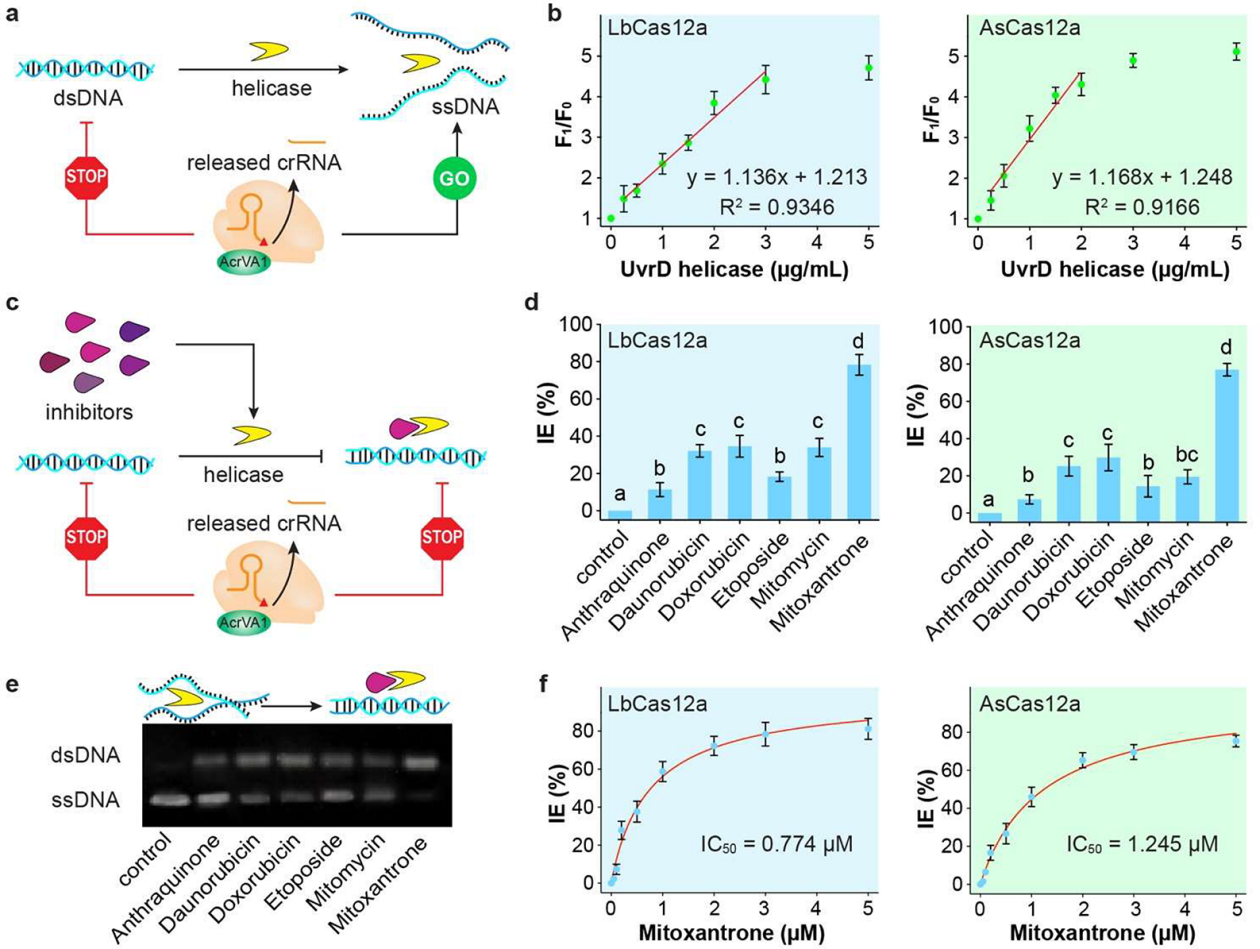
(a) Schematic illustration of the AcrVA1-assisted CRISPR biosensor to monitor the enzymatic activity of UvrD helicase. (b) Relative fluorescence intensity of the AcrVA1-assisted CRISPR biosensors toward the UvrD helicase at concentrations ranging from 0 to 5 μg/mL. (c) Schematic illustration of AcrVA1-assisted CRISPR biosensor to screen the inhibitors of the UvrD helicase. (d) Inhibition efficiency (IE) of different quinone derivatives to the UvrD helicase. (e) Agarose gel electrophoresis to characterize the IE of different quinone derivates of the UvrD helicase. (f) IE toward the concentration of mitoxantrone. All experiments were performed with at least three replicates, and the error bars represent the standard deviation.

Helicase, which is closely associated with cell growth and proliferation, has been previously suggested as the potential target for antiviral and anticancer interventions.^38^ Quinone-based synthetic compounds have been used to treat diseases by inhibiting the enzymatic activity of helicase. Thus, there is a crucial need for the rapid evaluation of the inhibition efficiency (IE) of helicase inhibitors, facilitating drug development. As shown in Figure 3c, helicase inhibitors can prevent helicase from unwinding dsDNA. There is no ssDNA produced or fluorescence signals observed from the AcrVA1-assisted CRISPR biosensor. In this study, we evaluated the inhibition efficiency of 6 quinone derivatives toward the UvrD helicase using the AcrVA1-assisted CRISPR-Cas12 biosensor, including anthraquinone, daunorubicin, doxorubicin, etoposide, mitomycin, and mitoxantrone. As shown in Figure 3d, all quinone derivatives can inhibit the enzymatic activities of helicase, and mitoxantrone reached an IE of as high as 80%. Meanwhile, we characterized the inhibition performances of the quinone derivatives toward the helicase using DNA gel electrophoresis (Figure 3e). All the quinone derivatives can stop helicase from unwinding dsDNA into ssDNA, with mitoxantrone exhibiting the most robust inhibition performance. All of these DNA gel results are consistent with the inhibition efficiency in Figure 3d.

Lastly, we studied the inhibition efficiency of mitoxantrone on the helicase enzymatic activity. For the AcrVA1-assisted CRISPR-Cas12a biosensor, the fluorescence intensities at the wavelength of 525 nm were significantly decreased upon increasing mitoxantrone concentrations (Figure S5). In addition, the IE increased rapidly with the mitoxantrone concentrations ranging from 0.05 to 2 μM and leveled off afterward. The half-maximal inhibitory concentration (IC_50_), which refers to the inhibitor concentration at which IE reaches 50%, is a crucial parameter for assessing the inhibitory capacity of the inhibitor.^39^ The IC_50_ values of mitoxantrone were calculated to be 0.774 and 1.245 μM for the two AcrVA1-assisted CRISPR-Cas12a systems, respectively (Figure 3f). These results indicate that our developed novel AcrVA1-assisted CRISPR detection assay is simple and feasible for monitoring helicase enzymatic activity and screening its inhibitors.

In summary, we have developed a novel AcrVA1-assisted CRISPR-Cas12a biosensor for the rapid and sensitive detection of ssDNA exclusively. In this detection strategy, AcrVA1 can inhibit the cleavage activity of the CRISPR-Cas12a biosensor by truncating its crRNA. Only ssDNA has the ability to recruit the AcrVA1-truncated crRNA fragment and recover the DNA cleavage activities of the CRISPR-Cas12a systems. The detection limits of ssDNA can be as low as 0.12 and 0.25 nM for the AcrVA1-assisted CRISPR-LbCas12a and CRISPR-AsCas12a biosensors, respectively. In addition, our developed anti-CRISPR-assisted biosensing system has been successfully applied to monitor the enzymatic activity of helicase and evaluate the inhibition performances of the helicase inhibitors. It is believed that our novel ssDNA detection system using an AcrVA1-assisted CRISPR biosensor holds great potential for helicase-related disease diagnosis and its inhibitory drug discovery.

## Supporting Information Available

Detailed experimental procedures; SDS-PAGE of purified proteins; the change of fluorescence intensity at 525 nm over reaction time; fluorescence spectra of the AcrVA1-assisted CRISPR biosensor to detect T-ssDNA; fluorescence spectra of the AcrVA1-assisted CRISPR biosensor to monitor the enzymatic activity of UvrD helicase; fluorescence spectra of the AcrVA1-assisted CRISPR biosensor to measure the UvrD helicase inhibition efficiency by mitoxantrone; DNA sequence used for fluorescence assay; and DNA used for AcrVA5 plasmid construction (PDF)
