# Supplementary material for: Novel Anti-CRISPR-Assisted CRISPR Biosensor for Exclusive Detection of Single-Stranded DNA (ssDNA)
Source: ACS Sens. 2024 Mar 5;9(3):1162–7. doi: 10.1021/acssensors.4c00201 (PMC10964243; doi:10.1021/acssensors.4c00201)
Supplement: Supplementary file 1 — se4c00201_si_001.pdf [file se4c00201_si_001.pdf]

Supporting Information

A novel anti-CRISPR-assisted CRISPR biosensor for exclusive detection of single-stranded DNA (ssDNA)

Qiaoqiao Ci[1], Yawen He[1], Juhong Chen[1,2*]

[1] Department of Biological Systems Engineering, Virginia Tech, Blacksburg, VA 24061, USA

[2] Department of Bioengineering, University of California Riverside, Riverside, CA 92521, USA

[*] Corresponding author:

Juhong Chen, Ph.D.

Assistant Professor

Department of Biological Systems Engineering, Virginia Tech, VA 24061, USA

Department of Bioengineering, University of California Riverside, CA 92521, USA

Email: jchen@ucr.edu

Experimental Procedures

1. Chemicals and materials

All plasmids were purchased from Addgene (Watertown, MA, USA). All DNA, RNA oligonucleotides, and ssDNA-FQ probes were commercially synthesized by Integrated DNA Technologies (IDT, Coralville, IA, USA), except the gene of AcrVA5, which was purchased from Twist Bioscience (South San Francisco, CA, USA) (**Table S1**). Microplate BCA^{TM} protein assay kit and protein concentrators (30,000 MWCO) were purchased from Thermo Fisher Scientific (Rockford, IL, USA). Tris-HCl, sodium chloride (NaCl), magnesium chloride ($MgCl_2$), nuclease-free water, terrific broth (TB), ampicillin, tris(2-carboxyethyl) phosphine (TCEP), dithiothreitol (DTT), isopropyl-β-d-thiogalactoside (IPTG), phenylmethylsulfonyl fluoride (PMSF), glycerol, lysozyme, sterile filter membrane, and adenosine triphosphate (ATP) were purchased from Fisher Bioreagents (Pittsburgh, PA, USA). Anthraquinone, daunorubicin, doxorubicin, etoposide, mitomycin, and mitoxantrone were purchased from Cayman Chemical Company Inc (Ann Arbor, MI, USA). Ni-NTA resin column, Heparin Hi-Trap, and Hi-Trap Q column were purchased from Neta Scientific Inc (Hainesport, NJ, USA). Plasmid Miniprep kit, Q5 High-Fidelity PCR kit, NEB buffer 2.1, UvrD helicase, and isothermal amplification buffer were purchased from New England Biolabs Ltd. (Ipswich, MA, USA).

2. Protein expression and purification (LbCas12a, AsCas12a, AcrVA1, AcrVA4, and AcrVA5)

Codon-optimized AcrVA5 gene was synthesized by Twist Bioscience and cloned into the pKEW189-MBP-TEV expression plasmid using standard molecular cloning technology, resulting in pKEW189-MBP-TEV-AcrVA5 plasmid. To express recombinant Cas12a nuclease and anti-CRISPR proteins, all plasmids (pMBP-LbCas12a, pMBP-AsCas12a, pKEW189-MBP-TEV-AcrVA1, pKEW212-MBP-TEV-AcrVA4, and pKEW189-MBP-TEV-AcrVA5) were transformed into *Escherichia coli* (*E. coil*) T7 express competent cells after the plasmid sequences were confirmed using Sanger sequencing.

Proteins were purified as described in a previously published study.[1] Briefly, *E. coil* T7 express cells containing Cas12a nuclease or AcrVA expression plasmids were grown in TB media with ampicillin (100 μg/ml) overnight. Overnight cultures were sub-cultured in TB media to an OD_{600} of 0.6 – 0.8, after which they were cooled on ice for 15 min before induction with IPTG (0.5 mM) at 16 °C for 16 h. Cells were harvested by centrifugation and resuspended in lysis buffer (50 mM Tris-HCl, pH 7.5, 500 mM NaCl, 1 mM TCEP, 0.5 mM PMSF, 5% (v/v) glycerol) supplemented with 0.25 mg/mL lysozyme. The cells were lysed by sonication at 20% amplitude for 20 min (3 sec on, 7 sec off), and the cell lysate was centrifuged at 7,500 rpm for 20 min at 4 °C. The supernatant was filtered using a 0.22 μm sterile filter membrane. Then,

the protein was purified over Ni-NTA resin in wash buffer (1x PBS, 300 mM NaCl, 25 mM imidazole, pH 7.5) and eluted in elution buffer (1x PBS, 300 mM NaCl, 250 mM imidazole, pH 7.5). Eluted proteins were digested using TEV protease at 4 °C for 2 days in TEV cleavage buffer (50 mM Tris-HCl, 0.5 mM EDTA, 1 mM DTT, pH 7.5). Digested proteins were loaded onto a Heparin Hi-Trap (Cas12a) or a Hi-Trap Q (AcrVA) and eluted over a salt gradient (20 mM Tris-HCl, pH 7.5, 1 mM TCEP, 5% (v/v) glycerol, 125 mM – 1 M KCl). The eluted proteins were concentrated and stored in 30-50% (v/v) glycerol at -20 °C until further use. The purity and integrity of the proteins used in this study were assessed by SDS–PAGE. The total protein concentration was measured using the Microplate BCA™ protein assay kit using BSA as the standard.

3. Preparation of dsDNA sample

Two complementary single-strand DNA (ssDNA) oligos (T-ssDNA and NT-ssDNA) were separately dissolved in nuclease-free water at a final concentration of 5 μM. The ssDNA solutions (10 μL for each) were mixed with 5x annealing buffer (10 μL, 300 mM KCl, 30 mM HEPES, 1.0 mM $MgCl_2$, pH 7.5) in a PCR tube. Nuclease-free water (30 μL) was added to reach a final volume of 50 μL. The solution was incubated for 3 min at 95°C and gradually/slowly cooled down to room temperature (over a period of 45 min). The dsDNA sample was stored at 4°C on ice until ready to use.

4. Fluorescence detection assay

Master mixture was prepared by mixing Cas12a nuclease, corresponding crRNA, AcrVA, and ssDNA-FQ probes in 1x NEB 2.1 buffer (50 mM NaCl, 10 mM Tris-HCl, 10 mM $MgCl_2$, 100 μg/mL BSA, pH 7.9) for 30 min. After adding DNA substrates (total 100 μL), the solutions were immediately added into a 96-well fluorescence plate, and incubated in a fluorescence plate reader (BioTek). The fluorescence intensities were measured (λex: 485 nm; λem: 525 nm) for 40 min at 37 °C. According to a previous study,[1] the Cas12a nuclease (5 nM), crRNA (6.25 nM), and ssDNA-FQ probes (10 nM) were first decided, and the concentration of remaining reagents was optimized based on the fluorescence readout. The F_1 represents the florescence intensity at the end point, while the F_0 represents the initial fluorescence intensity.

5. Helicase monitoring assay

The total reaction volume was 20 μL. UvrD helicases at different concentrations were first mixed with dsDNA substrate (200 nM) and ATP (5 mM) in the helicase assay buffer (20 mM Tris-HCl, 10 mM $MgCl_2$, 0.1 mg/ml BSA, pH 7.5). After incubation at 65 °C for 10 min, EDTA solution (10 mM) was added to stop the reaction. The reaction solutions (5 μL) were added into a 100 μL pre-assembled detection system (5 nM Cas12a and 6.25 nM crRNA with 30 nM AcrVA1 and 10 nM ssDNA-FQ probes in 1X NEB 2.1 buffer). The mixed reaction solution was

incubated in a fluorescence plate reader (BioTek) for 40 min at 37 °C, and the fluorescence spectra were recorded with excitation at 525 nm (λex = 485 nm).

6. Inhibitor screening

Helicase inhibitors at various concentrations and UvrD helicase (3 μg/mL) were pre-incubated in the helicase assay buffer. The dsDNA substrate (200 nM) and ATP (5 mM) in the helicase assay buffer were added to the reaction solution to obtain a reaction volume of 20 μL. The mixture was incubated at 65 °C for 10 min, and then EDTA solution (10 mM) was added to stop the reaction. The mixed solution was added to the AcrVA1-assisted CRISPR-Cas12a system (5 nM Cas12a, 6.25 nM crRNA, 30 nM AcrVA1, and 10 nM ssDNA-FQ probes in 1X NEB 2.1 buffer). The change of fluorescence intensity at 525 nm was recorded after incubation for 40 min at 37 °C (λex =485 nm). The reaction products were analyzed by electrophoresis on a 3% agarose gel.

The inhibition efficiency (IE) is defined as:

$$IE = 100\% \times [FL_{(\text{no inhibitor})} - FL_{(\text{inhibitor})}] / [FL_{(\text{no inhibitor})} - FL_0]$$

Where $FL_{(\text{no inhibitor})}$ and $FL_{(\text{inhibitor})}$ are the fluorescence intensities at 525 nm after 40 min of the UvrD helicase enzymatic reaction in the absence or presence of an inhibitor. FL_0 is the fluorescence intensity of the blank sample.[2]

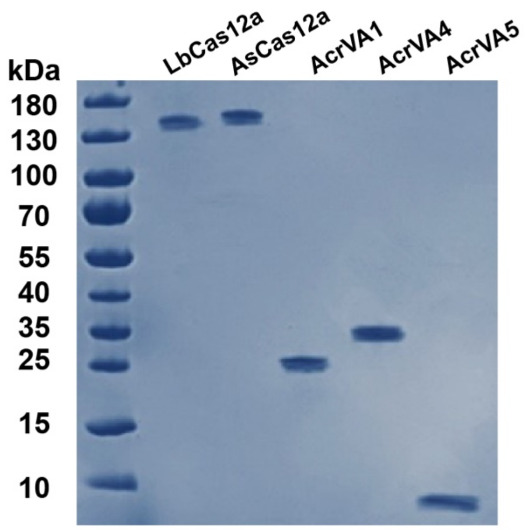

Figure S1. SDS-PAGE of purified two Cas12a nucleases and three anti-CRISPR type V-A proteins (AcrVAs), including LbCas12a, AsCas12a, AcrVA1, AcrVA4, and AcrVA5 (from left to right).

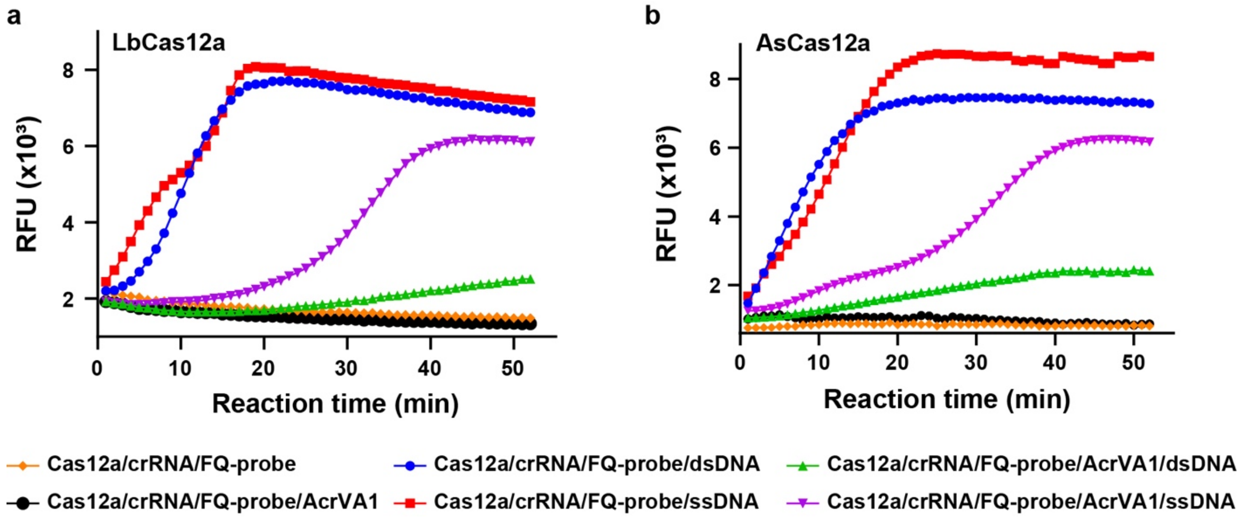

Figure S2. The change of fluorescence intensity at 525 nm over reaction time for Cas12a + crRNA + FQ probe (orange), Cas12a + crRNA + FQ probe + AcrVA1 (black), Cas12a + crRNA + FQ probe + dsDNA (blue), Cas12a + crRNA + FQ probe + ssDNA (red), Cas12a + crRNA + FQ probe + AcrVA1 + dsDNA (green), and Cas12a + crRNA + FQ probe + AcrVA1 + ssDNA (purple).

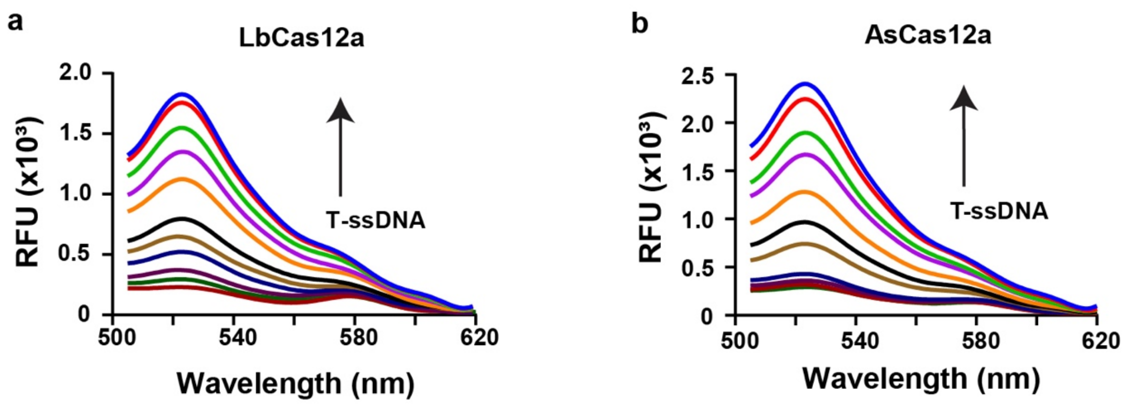

Figure S3. Fluorescence spectra of the AcrVA1-assisted CRISPR biosensor to detect T-ssDNA at different concentrations ranged from 0 to 200 nM, including 0, 0.5, 1, 2, 5, 10, 20, 50, 100, 150, and 200 nM.

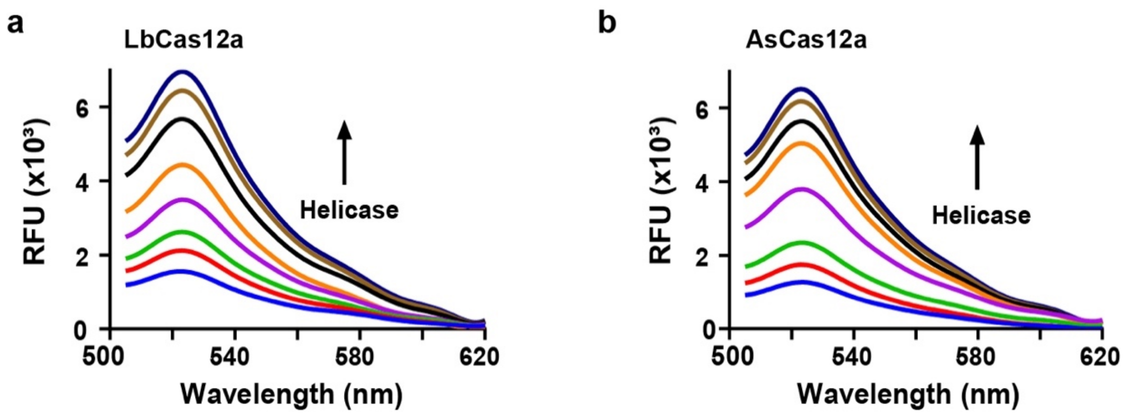

Figure S4. Fluorescence spectra of the AcrVA1-assisted CRISPR biosensor to monitor the enzymatic activity of UvrD helicase at different concentrations ranging from 0 to 5 μg/mL, including 0, 0.25, 0.5, 1, 1.5, 2, 3, and 5 μg/mL.

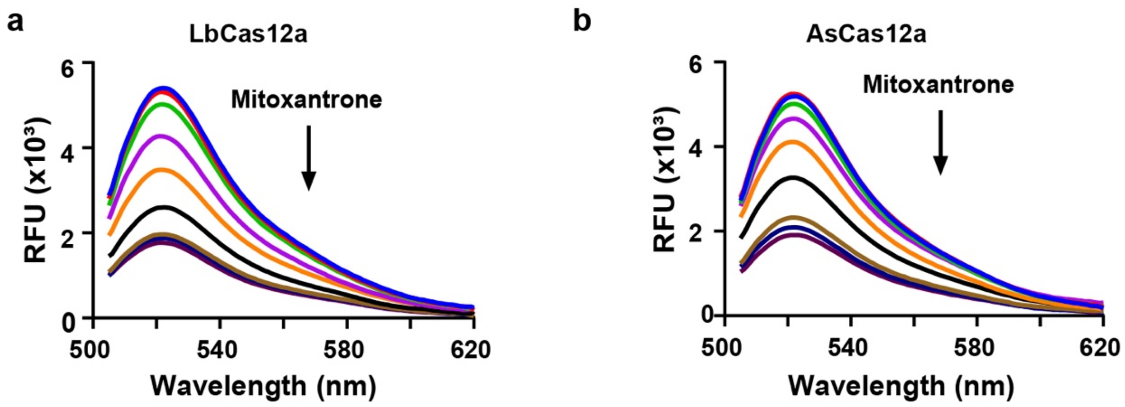

Figure S5. Fluorescence spectra of the AcrVA1-assisted CRISPR biosensor to measure the UvrD helicase inhibition efficiency by mitoxantrone at different concentrations ranging from 0 to 5 μM, including 0, 0.05, 0.1, 0.2, 0.5, 1, 2, 3, and 5 μM.

Supporting table

Table S1. The DNA sequence used for fluorescence assay in this study.

Name	Sequence (5'-3')
crRNA (LbCas12a)	AAUUUCUACUAAGUGUAGAUGAUCGUUACGCUAACUAUGA
crRNA (AsCas12a)	AAUUUCUACUCUUGUAGAUGAUCGUUACGCUAACUAUGA
Target ssDNA	TAGCATTCCACAGACAGCCCTCATAGTTAGCGTAACGATCTAAAGTTTTGTCGTC
Non-target ssDNA	GACGACAAAACTTTAGATCGTTACGCTAACTATGAGGGCTGTCTGTGGAATGCTA
ssDNA-FQ probes	/56-FAM/TTATT/3IABkFQ/

Table S2. The DNA used for AcrVA5 plasmid construction in this study.

Name	Sequence (5'-3')
Forward Primer (insert)	CTTCCAATCCATGAAAATCGAACTATCAGGCGGATATATCT
Reverse Primer (insert)	ACTTCCAATATTTAGCTCCATCTCATCAAGC
Forward Primer (Vector1)	GATGCGGCGGGACCAGAGA
Reverse Primer (Vector1)	CGATTTTCATGGATTGGAAGTACAGGTTTTCCT
Forward Primer (Vector2)	GATGGAGCTAAATATTGGAAGTGGATAACGGATCCGC
Reverse Primer (Vector2)	TGGTCCCGCCGCATCCATAC
AcrVA5 Fragment	CTTCCAATCCATGAAAATCGAACTATCAGGCGGATATATCTGCTACAGCATT<br>GAAGAAGATGAAGTTACCATTGACATGGTAGAGGTTACCACCAAACGTCAAG<br>GCATTGGCAGTCAGCTAATTGATATGGTTAAGGATGTAGCCCGTGAAGTTGG<br>CTTGCCAATTGGTCTATATGCCTACCCCCAAGATGACAGCATTAGCCAAGAA<br>GACTTGATTGAGTTTTATTTTTCTAATGACTTTGAGTATGACCCAGATGATGT<br>AGATGGTCGCTTGATGAGATGGAGCTAAATATTGGAAGT

References

[1] G. J. Knott, B. W. Thornton, M. J. Lobba, J.-J. Liu, B. Al-Shayeb, K. E. Watters, J. A. Doudna, *Nature structural & molecular biology* **2019**, *26*, 315-321.

[2] Q. Wang, K. Tan, H. Wang, J. Shang, Y. Wan, X. Liu, X. Weng, F. Wang, *Journal of the American Chemical Society* **2021**, *143*, 6895-6904.
